# Antimicrobial and physical properties of experimental endodontic sealers containing vegetable extracts

**DOI:** 10.1038/s41598-021-85609-4

**Published:** 2021-03-19

**Authors:** Daniela Coelho dos Santos, Andressa da Silva Barboza, Lara Rodrigues Schneider, Carlos Enrique Cuevas-Suárez, Juliana Silva Ribeiro, Melissa Feres Damian, Angela Diniz Campos, Rafael Guerra Lund

**Affiliations:** 1 Post-Graduate Program in Biochemistry and Bioprospecting, Federal University of Pelotas, Eliseu Maciel Avenue, Building 31, Pelotas, RS 96010-900 Brazil; 2 Post-Graduate Program in Dentistry, Pelotas Dental School, Federal University of Pelotas, 457 Gonçalves Chaves, Room 503, Pelotas, RS 96015-560 Brazil; 3 Dental Materials Laboratory, Academic Area of Dentistry, Autonomous University of Hidalgo State, Circuito Ex Hacienda La Concepción S/N, 42160 San Agustín Tlaxiaca, Hidalgo Mexico; 4 Brazilian Agricultural Research Corporation, Embrapa Temperate Climate, Highway BR-392, 78th km, 9th District, Monte Bonito, Pelotas, RS 96010-971 Brazil; 5 Laboratory of Oral Microbiology, Department of Restorative Dentistry, Pelotas Dental School, Federal University of Pelotas, Gonçalves Chaves Street, 457/Rm 702-3, Pelotas, RS 96015-560 Brazil

**Keywords:** Endodontics, Dentistry, Dental materials, Composite resin, Microbiology, Health care

## Abstract

To assess the antimicrobial activity and the physical properties of resin-based experimental endodontic sealers with the incorporation of vegetable extracts obtained from *Bixa orellana*, *Mentha piperita*, and *Tagetes minuta* species. The extracts were obtained and characterized by gas chromatography-mass spectrometry (GC–MS), and minimum inhibitory concentration (MIC) against *Streptococcus mutans*, *Enterococcus faecalis*, and *Candida albicans*. The extracts were individually incorporated into a dual-cure experimental sealer at a mass concentration of 0.5%. A commercial reference RealSeal was used. The sealers were evaluated by measuring the setting time, degree of conversion, dimensional stability, radiopacity, flow, and film thickness of these materials, also and its antimicrobial effect was evaluated using the direct contact test. Data were statistically analyzed by analysis of variance and Tukey’s post-hoc test at α = 0.05 significance level. The physical properties were not influenced by the addition of the vegetable extracts (*p* > 0.05). For *S. mutans*, only *T. minuta* and *B. orellana* groups presented antibacterial activity after 24 h of contact (*p* < 0.05). All extracts evidenced an antibacterial effect against *E. faecalis* (*p* < 0.05). The experimental sealers hold promise as a novel vegetable sealer with great antimicrobial activity and also great physical–mechanical properties. Nonetheless, more studies are needed.

## Introduction

Endodontic treatment has the main objective to eliminate the bacteria and their products from the root canal system to prevent reinfection^1–3^. To achieve this, a clinical protocol including chemomechanical preparation and adequate filling of root canal is performed^4^. Although these procedures allow a substantial microbial reduction, evidence suggests that persistent microorganisms after endodontic treatment can survive, interfering with the healing and repair processes^2,5^.

Despite the importance of clinical restorative protocol and material selection in the success of the endodontic treatment, the use of root canal sealers with antibacterial properties has been suggested for the reduction and elimination of persistent microorganisms^3^.

In this context, plants with various chemical constituents offer certain promising sources of new antimicrobial agents with general as well as specific activities^1,3,6,7^. For example, peppermint oil derived from *Mentha piperita* L. (Lamiaceae) has been commonly used in several pharmaceutical and industrial products due to its wide range of demonstrated pharmacological properties including antioxidant, antitumor, antiallergenic, antiviral, fungicide, insecticide, and antibacterial^8,9^. Furthermore, the powders and extracts of *Tagetes* species (Asteraceae) present insecticidal, antioxidant, and antibacterial properties^10,11^. Another example is found in *B. orellana*, which possesses antimicrobial, antioxidant, antidiabetic, anticonvulsant, and cardio-protective activity^12,13^. Essential oils and plant tinctures are examples of vegetable extracts obtained from different processes of chemical extraction of bioactive agents from plants. The essential oil of herbs is traditionally obtained by hydrodistillation or solvent extraction. Tinctures or also popularly known as simply ‘extracts’ are obtained through cold-pressing or soaking a plant (typically in water, oil, or alcohol) to create a tincture-type liquid^14,15^.

Considering the antibacterial potential of these species, this study aimed to evaluate the antimicrobial activity of essential oils from *M. piperita* and *T. minuta*, and the tincture of *B. orellana* by incorporating them into experimental root canal sealers in order to develop new products of dental use with antimicrobial activity against microorganisms that cause infections of the canal root system. Thus, it is extremely important to validate that the addition of the essential oils and tincture to root canal sealers neither damage the integrity of the root filling nor cause deterioration of the endodontic sealer. The null hypothesis is that the incorporation of vegetable extracts into the experimental root canal sealers will neither present antibacterial activity nor physicochemical changes in the endodontic sealers.

## Materials and methods

### Experimental design

This in vitro study was divided in two parts: (1) selection and characterization of essential oils from *M. piperita* and *T. minuta* and ethanolic tincture from seeds of *Bixa orellana* was performed to determine their essential constituents and minimum inhibitory concentration (MIC) against, *E. faecalis, S. mutans* and *C. albicans*; and (2) characterization of the resin-based experimental endodontic sealers after incorporation of the vegetable extracts throughout the direct contact test, flow, film thickness, dimensional stability, setting time, degree of conversion, and radiopacity.

### Selection and characterization of essential oils and tincture

#### Plant material

Aerial parts of *M. piperita*, *T. minuta,* and seeds of *B. orellana* were collected from the Brazilian Agricultural Research Corporation, Embrapa Temperate Climate, Monte Bonito, RS, Brazil. The essential oils from *M. piperita* and *T. minuta* were collected according to the National Sanitary Surveillance Agency-Anvisa^16^, using a *Clevenger* type apparatus for 3 h. The tincture of the annatto seeds from *B. orellana* was obtained via the technique described by Lorenzi and Matos^17^, with modifications, where 100 g of seeds were placed in 1000 mL of ethanol at 70% v/v. After 15 days, the tincture was filtered to separate the supernatant from the residue, which was then lyophilized for obtaining the dried extract. The tincture then was stored in a desiccator protected from light until its further use.

#### Determination of the core constituents of essential oils and tincture

Identification of the *T. minuta* and *M. piperita* essential oils compounds was performed using a gas chromatograph-mass spectrometer GC/MS-QP2010 SE (Shimadzu, Japan). Separation of the compounds was done on a RTX-5MS capillary column (30 mm × 0.25 mm, 0.25 μm; Restek, USA). The compounds were quantified by their normalized area and were identified by the mass spectra using the GC Solution Program and the NIST 8 library.

The *B. orellana* tincture compounds were identified according to Swain and Hillis^18^, with minor modifications. A UV/Vis spectrophotometer (JENWAY 6705, Cole-Parmer, UK) at 725 nm was used for the quantification of the phenolic compounds. A standard curve prepared with gallic acid was used, and the results were expressed in milligrams of gallic acid equivalent. For determining the carotenoids, the method described by Rodriguez-Amaya^19^, was followed with minor modifications, the reading was carried out in a UV/Vis spectrophotometer (JENWAY) at 470 nm, and the results were expressed in microgram of sample lycopene/g.

#### Antimicrobial assay

The reference strains used in this study were selected based on their pathological effects on dentistry. The strains were *E. faecalis* American Type Culture Collection (ATCC) 4083*, S. mutans* ATCC 25175, *and C. albicans* ATCC 62342*.* The microorganisms used in this study were collected from the Research Laboratory of Microbiology of Dentistry School, Federal University of Pelotas (UFPel).

#### Determination of MIC

This test evaluated the antimicrobial activity from the essential oils of *T. minuta* and *M. piperita* and the ethanolic tincture from seeds of *B. orellana*. The MIC was determined in triplicate by the broth microdilution technique (MIC) using a modified version of the reference documents M27-A3^20^, and M7-A7^21^, adapted for use with vegetable extracts. In order to standardize the inoculum, culture suspensions were prepared, diluted in 0.9% saline using a 0.5 MacFarland scale to obtain approximately 1.5 × 10^8^ Colony Forming Units (UFC/mL) for bacteria and 2.0 × 10^6^ CFU/mL for yeast. The susceptibility test was performed on 96-well microplates; essential oils and tincture were dissolved in ethanol at 0.5 g/mL concentration. The solution was placed into 96-well plates comprising the culture medium (RPMI for fungi and Mueller-Hilton for bacteria) in concentrations ranging from 0.39 to 50 mg/mL (*B. orellana* tincture) and from 0.10 to 25 μL/mL (*T. minuta* or *M. piperita* essential oils). Furthermore, the aerobic and microaerophilic bacteria were incubated for 24 h, and fungi for 48 h. *S. mutans* was incubated in microaerophilic conditions. Microplate wells with only the essential oils or tincture with culture medium were used as a negative control, whereas cavities comprising only the microbial suspensions were used as a positive control. Three replicates were made for each concentration of the tested vegetable extracts for the MIC assay, in each experiment. The experiment was repeated three times.

### Characterization of the resin-based experimental endodontic sealers

#### Formulation

The experimental dual-cured resin-based endodontic sealers were formulated as two paste materials. The compositions of the experimental materials are summarized in Table 1. All the materials were formulated by mixing the components using a high-speed mixer SpeedMixer DAC 150.1 FV (FlackTek Inc., UK).

**Table 1:** Composition of the experimental endodontic sealers.

Materials	% weight
**Base paste**	
Ethoxylated bisphenol-A dimethacrylate 30	40
Exothane 8	10
Polyethylene glycol 400 dimethacrylate	10
Triethylene glycol dimethacrylate	10
Camphorquinone	0.4
Ethyl 4-(dimethylamino)benzoate	0.6
Aerosil 380	4
Ytterbium fluoride	25
**Catalyst paste**	
Ethoxylated bisphenol-A dimethacrylate 30	40
Exothane 8	20
Triethylene glycol dimethacrylate	10
Benzoyl peroxide	0.4
p-Toluenesulfonic acid	0.6
Aerosil 380	4
Ytterbium fluoride	25

Three different base pastes were prepared by adding *T. minuta* (G1), *M. piperita* (G2), and *B. orellana* (G3) at mass concentrations of 0.5 wt%. This concentration was selected according to previous studies^8,13^ and a previously screened concentration test, through the MIC and degree of conversion tests. Being chosen the concentration that did not inhibit polymerization and at the same time had antimicrobial action. This concentration was higher than the MIC for the three strains *E. faecalis, S. mutans* and *C. albicans.* A base paste without essential oils or tincture addition was used as a control. The physical properties of the endodontic sealers were evaluated according to the ISO 6876 guidelines (ISO 2012)^22^.

#### Flow

After mixing the experimental materials, 0.05 mL (± 0.005) of each sealer was dispensed on a glass slab (40 × 40 × 5 mm), and after mixing for 3 min, another slab with a mass of 20 g (± 2) and a load of 100 g was placed on the top of the material. Then, after 7 min, the load was removed and the material was photoactivated for 20 s. The major and minor diameters of the compressed material were measured. The test was conducted in triplicate for each group (*n* = 3).

#### Film thickness

The film thickness was determined by the difference in the thickness of the plates with and without the sealer. Two glass slabs with a surface area of 200 mm^2^ and 5.0 mm thickness were placed together and their thickness was measured. After mixing, 0.05 mL of each experimental endodontic sealer was placed on the surface of the first slab, and the second slab was placed on the top of the material. After mixing for about 3 min, a load of 150 N was applied on top of the glass slab. Then, after 7 min, the load was removed and the material was photoactivated for 20 s. After the photopolymerization stage, the thickness of the two glass plates and the interposed sealer film were measured. The test was carried out in triplicate for each group (*n* = 3).

#### Dimensional stability

The cylindrical specimens (3.5 mm height × 3 mm diameter) were fabricated employing a silicon mold (*n* = 8). After removing from the mold, flat surfaces of each specimen were polished with a 600-grit wet sandpaper and its initial length was measured with a digital caliper (Mitutoyo Sul Americana Ltda, Santo Amaro, São Paulo, Brazil). Thereafter, the specimens were stored in flasks containing distilled water at 37 °C for 30 days. After the storage time, they were removed from the flasks, dried, and their final lengths were measured. The percentage of dimensional change was calculated as follows according to Carvalho-Junior et al.^23^:

$$DS=\left[\left(L30-L\right)/L\right]\times 100$$

where L is the initial length of the specimen, and L30, the length after 30 days.

#### Setting time

The experimental endodontic sealers were mixed and inserted into stainless-steel molds (10 mm diameter × 1 mm height). The determination of setting time was performed using an indenter with a head weight of 200 g. During each hour, the indenter was carefully lowered vertically on to the horizontal surface of the sealer. The setting time of each sealer was established by calculating the mean time elapsed from mixing until the indenter failed to leave an indentation on the surface of the specimens. During all the procedure, the mixture was maintained into a dark environment for avoiding light-activation of the material; under these conditions, only chemical activation is expected.

#### Degree of conversion

The degree of conversion of the experimental resin sealers (*n* = 3) was evaluated using FTIR spectroscopy (RT-FTIR Shimadzu Prestige 21 Spectrometer, Shimadzu, Japan) with an attenuated total reflectance device. The experimental endodontic sealers were mixed and a small sample (~ 100 µg) of the mixture was placed on the diamond cell window of the ATR unit. A spectrum was obtained before and after the material was irradiated for 20 s with a photopolymerization light-emitting diode (LED) unit. An infrared spectrum of the uncured and cured materials was obtained. The height of the aliphatic C=C peak absorption at 1638/cm and the aromatic C=C peak absorption at 1609/cm, for each spectrum. The aromatic C=C vibration was used as an internal reference. The double bond conversion was determined by the following equation^24^:

$$double\,bond\,conversion \left(\%\right)=100\left[1-\frac{\left(\frac{{A}_{1638}}{{A}_{1609}}\right)polymer}{\left(\frac{{A}_{1638}}{{A}_{1609}}\right)monomer}\right]$$

#### Radiopacity

For evaluation of radiopacity, the materials were mixed and poured into a circular metallic matrix with 5 mm of internal diameter and 1 mm of thickness (*n* = 5), covered with polyester strips and photoactivated for 20 s on each surface. The sealers samples were positioned on occlusal phosphor plates of the VistaScan Plus digital system (Dürr Dental AG, Bietigheim-Bissingen, Germany) and radiographed with a X-ray unit (Ion 70x, Procion, Ribeirão Preto, São Paulo, Brazil) with 70 kVp, 8 mA, exposure time of 0.2 s and a focal length of 40 cm. An aluminum step wedge, with purity greater than 98%, measuring 50 × 20 mm and thickness varying in step form every 1 mm was used as a reference. The five samples of each group experimental and the aluminum step wedge were placed on the occlusal phosphor plates and five radiographs were taken and processed with the VistaScan Plus software (DBSWIN Imaging Software, Dürr Dental AG, Bietigheim-Bissingen, Germany). Three measures were performed in each sample.

#### Antimicrobial effect of the experimental sealers—direct contact test

The direct contact test was performed according to a method previously described by Zhang et al.^25^. Cylindrical samples (7 mm diameter, 1 mm thickness; *n* = 5) of experimental endodontic sealers were prepared by filling the uncured materials into silicon molds. Thereafter, the samples were irradiated using a commercial LED unit (Ultra Radii, SDI, Australia) for 20 s on both sides. After polymerization, the samples were sterilized by gamma radiation using a total dose of 4.08 kGy.

The surfaces of the materials disks were inoculated with 20 μL of microbial suspension (approximately 1 × 10^6^ cells) and were incubated for 1 h or 24 h at 37 °C in a moist atmosphere into a 96-well microplate. Thereafter, 180 μL of culture medium (BHI for *S. mutans* and *E. faecalis* and, Sabouraud’s Dextrose [SD] for *C. albicans*) broth was added to each well and shaken for 10 min. Microbial suspensions were serially diluted, plated into disposable Petri dishes containing BHI and SD, and were incubated at 37 °C for 24 h. After incubation, the colonies on the plates were enumerated. The viable bacteria count (CFU) was converted to log^10^ values, and the results were expressed in CFU/mL. The test was performed in triplicate.

### Statistical analysis

The statistical analysis was analyzed using IBM SPSS Statistics software (v 20.0; IBM, USA). The data were evaluated to check the distribution normality and variance homogeneity. Analysis of variance (ANOVA) was used to evaluate the effect of the independent variable (material) on the flow, film thickness, dimensional stability, and degree of conversion. Data from direct contact test was transformed by Log^10^ and then subjected to ANOVA on ranks test. The significance level was chosen at *p* < 0.05.

## Results

### Determination of the core constituents of essential oils and tincture

Results from the gas chromatography-mass spectrometry (GC–MS) analysis of essential oil of *M. piperita* and *T. minuta* are presented in Table 2. Fifteen compounds were identified for the *T. minuta* essential oil, of which, (74.38%) were d-carvone compounds. For the *M. piperita*, 12 compounds were identified, the most abundant being the Trans-beta Ocimene (30.47%) and Cis-Tagetone (18.81%). For *B. Orellana* tincture, the essential components identified were carotenoids and phenolic compounds.

**Table 2:** Identification of the compounds (%) of the essential oils of *M. piperita* and *T. minuta,* and crude ethanolic tincture of *B. orellana.*

Chemical compound	*M. piperita*	*T. minuta*
Anethol	–	1.06
Alpha.-caryophyllene	–	0.98
Alpha-humulene	0.66	–
Beta-cis ocimene	0.61%	–
Caryophyllene	–	3.96
Cis-carveol	–	2.27
Cis-tagetone	18.81%	–
d-Carvone	2.46%	74.38
Dihydrocarveol	–	4.61
Dihydrocarvone	–	1.56
d-Limonene	7.41%	1.35
Elemol	–	0.73
Elixene	0.57	–
Germacrene B	0.65%	1.46
Myrtenal, dihydro	0.49%	–
ND	–	1.71
ND	–	0.92
ND	21.71	–
ND	0.61	–
ND	1.62	–
ND	1.79	–
ND	1.19	–
ND	1.14	–
Pulegone	–	0.57
Terpene-4-ol	–	3.36
Trans-beta ocimene	30.47%	–
Trans-carveol	–	1.08
Trans-tagetone	7.55%	–
3-7-Dimethyl 2-3ª4,5,6-hexahydro-1-benzofuran	1.31%	–
3-Ethoxy-4-methoxyphenol	0.95%	–
***Bixa orellana***		
Carotenoids	276.47 mg/100 g	In β-carotene
Phenolic compounds	44.3 mg/g	In acid gallic

*ND* not identified.

### Antimicrobial assay—determination of MIC

Based on the MIC evaluation, the strains used in this test were sensitive to *B. Orellana* tincture and *M. piperita* and *T. minuta* essential oils. *S. mutans, E. faecalis,* and *C. albicans* were sensitive to the minimal concentrations used in this test (Table 3). The minimum inhibitory concentration (MIC) of *B. orellana* tincture was 3.12–6.25 mg/mL against bacteria and 50 mg/mL against *C. albicans* while the MIC of *M. piperita* and *T. minuta* oils was 0.1–12.5 µL/mL against bacteria and 0.25–6.25 µL/mL against *C. albicans*.

**Table 3:** In vitro antimicrobial activity of the essential oils and vegetable extracts by determining the minimum inhibitory concentration (MIC).

Natural product	Microorganisms tested		
	*C. albicans*	*E. faecalis*	*S. mutans*
*B. orellana* (seed)	50 mg/mL	6.25 mg/mL	3.12 mg/mL
*M. piperita* (leaves)	0.25 µL/mL	0.10 µL/mL	0.10 µL/mL
*T. minut*a (leaves)	6.25 µL/mL	6.25 µL/mL	12.5 µL/mL

### Physical properties of the resin-based experimental endodontic sealers

Table 4 presents the physical properties of experimental endodontic sealers. Considering the mean values, the greatest flow was observed for the *T. minuta* group, whereas the lowest was observed for Real Seal. All experimental sealers were under the specifications provided by ISO 6876^22^, except for film thickness values, which were higher than the value indicated (50 µm).

**Table 4:** Physicochemical and mechanical properties evaluated in different experimental endodontic sealers.

Group	Flow (mm)	Film thickness (µm)	Dimensional stability (%)	Setting time (h)	Degree of conversion (%)
*T. minuta*	21.06 (0.18)^a^	100.00 (0.00)^a^	8.71 (3.02)^a^	48	97.83 (0.41)^a^
*M. piperita*	20.86 (0.23)^a^	83.33 (11.54)^a^	8.38 (3.43)^a^	48	87.89 (0.86)^b^
*B. orellana*	20.96 (0.23)^a^	106.66 (15.28)^a^	8.00 (2.27)^a^	48	90.08 (0.20)^b^
Control	20.95 (0.11)^a^	83.33 (5.77)^a^	7.44 (1.48)^a^	40	74.53 (0.91)^c^
RealSeal™	20.93 (0.20)^a^	36.67 (5.77)^b^	6.00 (2.68)^a^	24	53.84 (2.52)^d^

Common corresponding letters (a–d) in a given column indicate no significant difference (p < 0.05).

**Figure 1:**
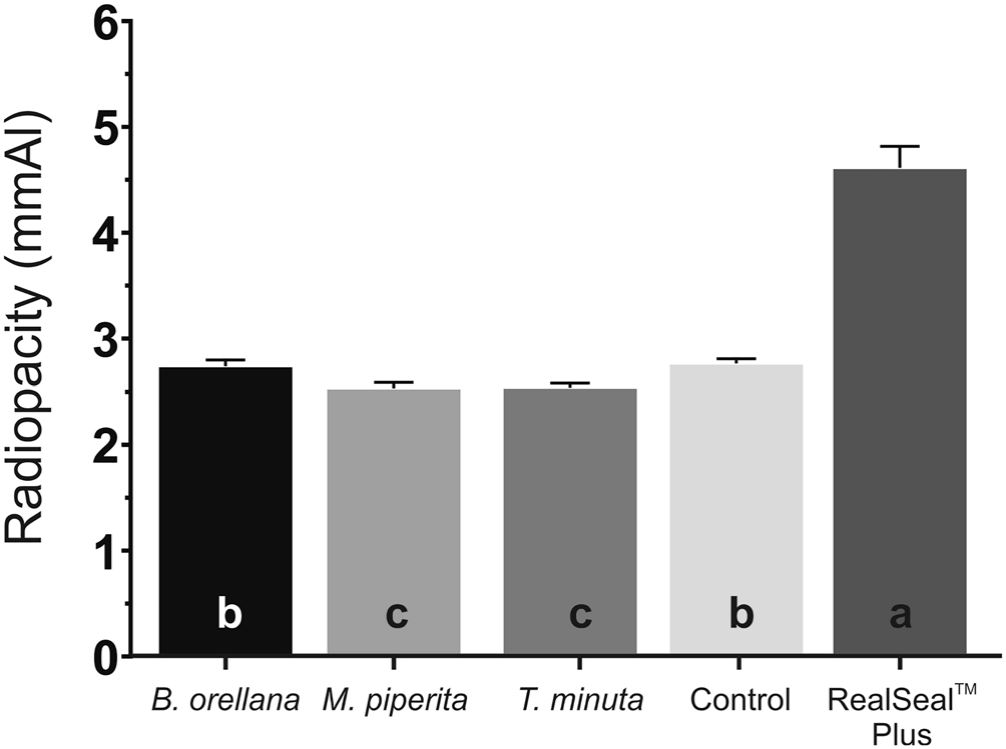
Radiopacity in mmAl of the endodontic sealers evaluated. Different lowercase letters indicate the presence of statistically significant differences (p < 0.05). Control group = Experimental endodontic sealer with no added vegetable extracts.

Figure 1 depicts that there was a statistically significant difference (*p* < 0.001) in the radiopacity of the various experimental materials evaluated. RealSeal Plus achieved the statistically significant higher radiopacity (p < 0.001). The base material and the material comprising *B. orellana* tincture attained higher values of radiopacity compared to the materials containing *M. piperita* and *T. minuta* essential oils; however, none of the experimental materials achieved the radiopacity value equivalent to 3-mmAl, which is the recommended minimum value for sealer materials, in accordance with the ISO 6876 specifications^22^.

### Antimicrobial effect of sealers formulations containing the vegetable extracts

**Figure 2:**
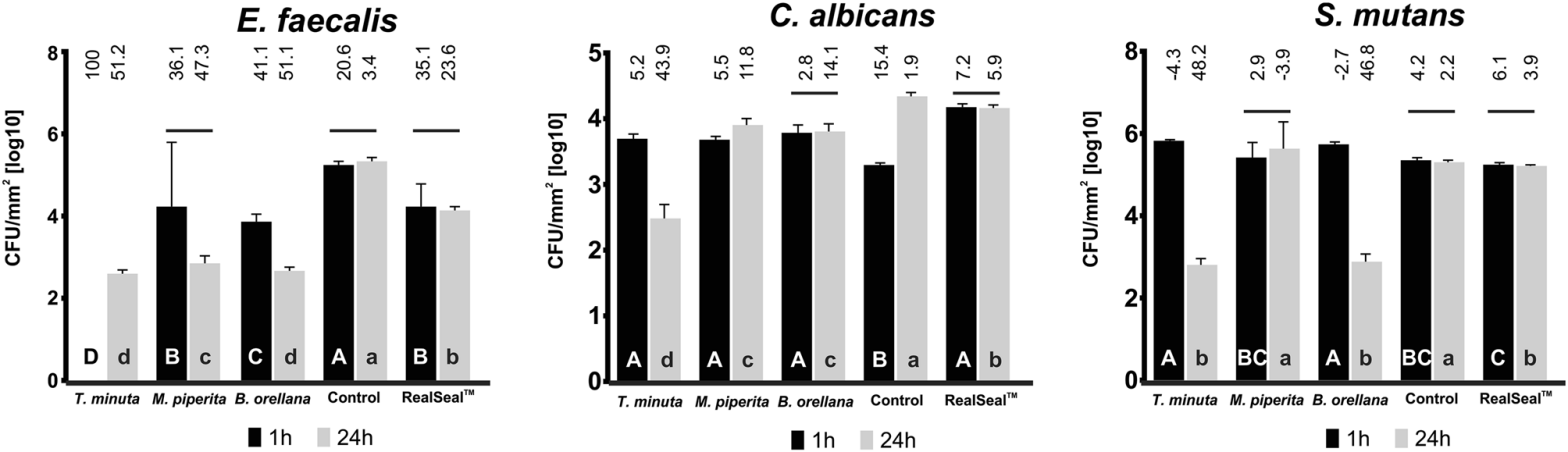
Antimicrobial effects of the test materials. Columns under the same horizontal line indicate no differences between different incubation times (1 h or 24 h). Uppercase letters indicate differences between endodontic sealers at 1 h of incubation time. Lowercase letters indicate differences between endodontic sealers at 24 h of incubation time. Numbers above columns indicate the percentage of log^10^ reduction when compared to the microbial growth. Control group = Experimental endodontic sealer with no addition of tincture or oils.

Considering the direct contact test, all experimental sealers with the incorporation of essential oils and tincture demonstrated antibacterial effect against *E. faecalis*. For this microorganism, *T. minuta* sealer was able to inhibit the total growth after 1 h of incubation (Fig. 2), and after 24 h of incubation, together with *B. orellana* sealer, demonstrated strong antibacterial activity. For *C. albicans, T. minuta* sealer, demonstrated antifungal activity after 24 h of incubation. *T. minuta* and *B. orellana* sealers presented considerable antibacterial effect after 24 h of incubation against *S. mutans*,

## Discussion

Residual microorganisms may lead to treatment failure in endodontic therapy. The use of a root canal sealer with good antimicrobial activity is essential for the long-term success of endodontic therapy^3^. Due to this, in the present study, the antimicrobial activity against *S. mutans*, *E. faecalis,* and *C. albicans* of experimental root canal sealers comprising tincture and essential oils from, respectively, *B. orellana*, *M. piperita,* and *T. minuta* was determined. According to the results, all materials exerted antibacterial activity, and therefore, the null hypothesis was rejected.

The major chemical components found in the essential oils of *M. piperita* and *T. minuta* were Trans-beta Ocimene and d-Carvone, respectively (Table 2). These results highlight the differences in concentrations of chemical components that may vary from plant to plant, as it is known that the chemical components of plants vary with plant maturity, species, geographical region, and processing conditions^10^. The method of extracting the seeds from B*. orellana* guarantees higher quantities of carotenoids and phenol groups^18^. In a recent study^26^, it was found that the antibacterial activity of *B. orellana* tincture is also due to flavonoids and alkaloids, the ability to complex proteins and bacterial and lipophilic cell walls, as well as rupture of bacterial membranes has been observed, recent studies have stated that the extracts contained many active phyto-constituents which can be responsible for their biological activity and that even at higher doses, it is safe and does not produce cytotoxicity^17,18^.

Concerning *M. piperita*, the studies demonstrated the inhibition of microorganisms such as *Escherichia coli, Staphylococcus aureus,* and *C. albicans* even at low concentrations. The antimicrobial activities of *M. piperita* and *T. minuta* have been attributed to the high levels of monoterpenes, thus exhibiting antimicrobial activity against Gram-positive and Gram-negative bacteria^8,10,27^. Although the antimicrobial activity of the tested compounds has already been well cited in the literature^9,28,29^ no study was found evaluating the antimicrobial activity of formulations of dental materials containing these vegetable extracts, such as an endodontic sealer.

Due to the polymeric nature of the endodontic sealers tested, the addition of non-polymerizable substances, like the vegetable extracts used in this study, could influence the final properties of the materials. As enhanced material properties are related to high-quality root filling, endodontic sealers must meet several requirements. In this sense, physical tests in this study were performed according to ISO 6876 specifications^24^, which ensure the reproducibility and allow us to comparing our experimental materials with others.

Considering the flow analysis, no statistical differences were observed among the materials, which allows to determine that the addition of vegetable extracts would not affect the capacity of the endodontic sealer to penetrate ramifications of the root canal system and dentinal tubules and small irregularities^30^. Also, worth is mentioning that all materials evaluated met the criteria established in ISO 6876^22^.

Film thickness provides information about the volume occupied by the endodontic sealer after filling in the root canal system^31^. In addition to provides a better seal, a thin film thickness is required to ensure an appropriate wetting of the dental substrate^32^. According to the results, the addition of *B. orellana, M. piperita,* or *T. minuta*, when compared to the model experimental sealer did not affect the film thickness; however, the values exceed is the specifications of ISO 6876 (50 µm)^22^. Presumably, other material characteristics, such as particle size distribution and viscosity, had higher influences on this property^33^.

According to the results, all materials underwent an expansion after water storage, which suggests highly hydrophilicity of the materials evaluated^34^. The organic matrix used for the formulation of the endodontic sealers included methacrylate monomers that contain a large amount of hydrophilic groups in their structure, which ones can form hydrogen bonds with water molecules, with the consequent expansion of the material^35^. Furthermore, the leakage of unreacted components, like the vegetable extracts, from the polymeric matrix could have contributed to promoting the expansion of the materials. Actually, a slight increase in the percentage expansion is observed in the experimental materials when compared to the control formulation, which indicates that, once the vegetable extracts are released, the space they leave within the polymeric matrix could be filled by water^36^. The expansion of the materials could improve the sealing ability of the materials evaluated^34^.

Setting time of endodontic sealers was also evaluated. The time for sealers to set is important clinically, enabling the placement of sealer in more than one canal^37^. In this study, the incorporation of vegetable extracts increased the setting time of the control formulation from 40 to 48 h. The increase in the setting time of the experimental materials could be due to the fact that vegetable extracts incorporated act as retarders in the polymerization reaction. This could be explained due to the presence of terpenoids within the vegetable extracts added into the formulation of the endodontic sealer, which could have increased the induction time of the polymerization reaction, resulting in a delay of the final set of the material^38^. Despite this, the delay in setting time observed for the experimental materials may favor their antibacterial activity that is mediated by substances released during the setting of the material^2^. However, the setting time of 48 h can be considered with caution, as chemical activation can occur in the deeper portions of the root canal and, therefore, this information cannot be translated directly into the clinical scenario to infer the release of the antimicrobial action. Also, the leaching of essential oils can be facilitated in a non-polymerized matrix; however, the release of cytotoxic compounds from the methacrylate structure is not desired, especially considering that some photoactivation would occur in the apical region close to the periapical tissue. Further studies are needed to verify if the depth of polymerization of these experimental materials interfere in the lixiviation or cytotoxicity of these endodontic sealers containing vegetable extracts^39–41^.

All experimental materials presented a higher degree of conversion than Real Seal. Also, the addition of vegetable extracts allowed to increase the degree of conversion values of the base formulation used as control. Two explanations may be suggested to explain this increase. First, as the addition of vegetable extracts into the endodontic sealers resulted in a delay of the polymerization rate reaction, it could be theorized that polymer vitrification was delayed too, facilitating the polymer chains formation with higher molecular weight and higher degree of conversion^38^. Alternatively, this increase could be explained due to the presence of terpenoids as main constituents of the vegetable extracts added into the experimental materials. This family of molecules possesses a carbon–carbon double bond, which is capable to undergo on a free radical polymerization, what allows it to copolymerize with another functional groups, like the methacrylates contained in the monomers from the organic matrix used here for the formulation of the materials^38,42^. A higher degree of conversion value for the resin-based endodontic sealers is essential as the presence of uncured material in the tooth apex could promote an inflammatory reaction, and consequently, a failure of the endodontic treatment^43^.

The endodontic sealer must present radiopacity to identify teeth with and without root canal treatment. According ISO 6876^22^, the endodontic sealers must have radiopacity equal to or greater than the equivalent radiopacity of 3-mmAl. In this study, the experimental base sealer without natural extract and the sealer with *B. orellana* presented the highest radiopacity values; however, they do not attain the radiopacity values recommended by ISO^22^. The Ytterbium fluoride promotes radiopacity to the experimental materials tested; however, the amount of Ytterbium fluoride incorporated in the sealer may be insufficient to provide adequate radiopacity to the experimental sealers.

The direct contact test is a quantitative and reproducible antimicrobial assay, which relies on direct contact to the test the microorganisms with the test material for a controlled period and independent of the diffusion and solubility properties of the material tested and media^16^. In this regard, *E. faecalis*, S. *mutans* and *C. albicans* was selected to evaluate the capacity of sealers to inhibit species related to endodontic failure. *E. faecalis* are Gram-positive bacteria known to be associated with secondary endodontic infection, and its persistence is credited with its capacity to colonize the root canal and resist treatment^44^. Another microorganism associated with apical periodontitis is the *S. mutans*. The *S. mutans* is found in infected root canals, having a potential role in the pathogenesis of endodontic infections^45^. Further, *C. albicans* has a major role in endodontic treatment failure as the most important fungus isolated from the root canal system^46^. Based on this in the present study these microorganisms were selected to comprise a primary evaluation of the antimicrobial activity of the experimental sealers the direct contact test. In our findings, all of the sealers revealed strong antibacterial activity. The *E. faecalis* was completely inhibited by *T. minuta* experimental sealer during 1 h exposure, however, in 24 h it showed growth. This justifies a bacteriostatic rather than bactericidal effect of this formulation against this strain. *T. minuta* also presented antifungal activity against *C. albicans,* and antibacterial activity against *S. mutans*. d-Carvone, the major component of *T. minuta*, has been proved to exert antibacterial and antifungal activity, and its mechanism of action is probably due to alteration of the outer membrane of the microorganism^47^. In the case of *B. orellana*, this sealer demonstrated strong antibacterial activity against *E. faecalis and S. mutans*, after 24 h of incubation. The main components detected in this vegetable extract were carotenoids and phenolic compounds, which has been proven to possess antibacterial activity^48^.

This work was an initial step for searching materials with antimicrobial potential for future use in endodontics. It is undeniable that there are some complexities involved in laboratory- and industry-based dental materials development process. For that reason, in recent years, the scientific community have expended great efforts to develop endodontic sealers possessing good mechanical and biological properties. In this way, future studies should focus on evaluating their biological response in cell lines as well as clinical studies are necessary to elucidate if these results are clinically relevant, although the incorporation of vegetable extracts improved the antimicrobial effect of the experimental sealers against *E. faecalis*, *S. mutans*, and *C. albicans*. Furthermore, it is also important to consider the limitations of the present in vitro study. Further studies are needed to identify and standardize the existing chemotypes for each of these plant species so that their use at the industrial level can be considered. It is important to develop analytical methodologies that can standardize the cultivation and chemical composition of these plant extracts to ensure the effectiveness of these compounds and their effective incorporation in dental materials. Further, more complex antimicrobial methodologies to better simulate oral conditions, such as microcosms biofilm or in vivo biofilm essays, needs to be explored. Moreover, the longevity of the antimicrobial and biological activity was not determined; perhaps establish the mechanisms of action and interaction of the material with the cells and microorganisms may elucidate better the presented results. Finally, the overall efficacy of the proposed technology needs to be assessed by animal models and clinical trials.

In summary, the incorporation of vegetable extracts at relevant concentrations demonstrated the antimicrobial potential of three different plant species in experimental endodontic sealers. *T. minuta,* and *M. piperita* essential oils and the crude ethanolic tincture of *B. orellana*, revealed similar sensitivity to microbial growth. Compared to the cement formulations containing the plant extracts, it was possible to identify the antimicrobial activity between the experimental groups tested, and all of them proved to be effective for the analyzed substrates; however, concerning time × effectiveness, *T. minuta* presented better results to be used as sealer for the root canals.
